# Supplementary material for: The Effect of Extraction Methods on Phytochemicals and Biological Activities of Green Coffee Beans Extracts
Source: Plants (Basel). 2023 Feb 6;12(4):712. doi: 10.3390/plants12040712 (PMC9966978; doi:10.3390/plants12040712)
Supplement: Supplementary file 1 [file plants-12-00712-s001.zip › plants-2142351-supplementary.pdf]

The Effect of Extraction Method on Phytochemicals and Biological Activities of Green Coffee Beans Extracts

Octavia Gligor [1], Simona Clichici [2], Remus Moldovan [2], Dana Muntean [3], Ana-Maria Vlase [1,*], George Cosmin Nadăș [4], Ioana Adriana Matei [4], Gabriela Adriana Filip [2,*], Laurian Vlase [3], and Gianina Crișan [1]

[1] Department of Pharmaceutical Botany, Iuliu Hațieganu University of Medicine and Pharmacy, 8 Victor Babeș Street, 400347 Cluj-Napoca, Romania
[2] Department of Physiology, Iuliu Hațieganu University of Medicine and Pharmacy, 8 Victor Babeș Street, 400347 Cluj-Napoca, Romania
[3] Department of Pharmaceutical Technology and Biopharmaceutics, University of Medicine and Pharmacy, 8 Victor Babeș Street, 400347 Cluj-Napoca, Romania
[4] Department of Microbiology, University of Agricultural Sciences and Veterinary Medicine, 3/5 Mănăștur Street, 400372 Cluj-Napoca, Romania
* Correspondence: gabriela.filip@umfcluj.ro (G.A.F.); gheldiu.ana@umfcluj.ro (A.-M.V.)

4. Materials and Methods

4.6. Chromatographic Analysis

The UV chromatograms of the polyphenolic compounds detected in the analyzed extracts are displayed in Figures S1–S11 (the UV detector operated at λ=330 nm and for detection of the flavonoids and their aglycones, the UV detector operated at λ=370 nm).

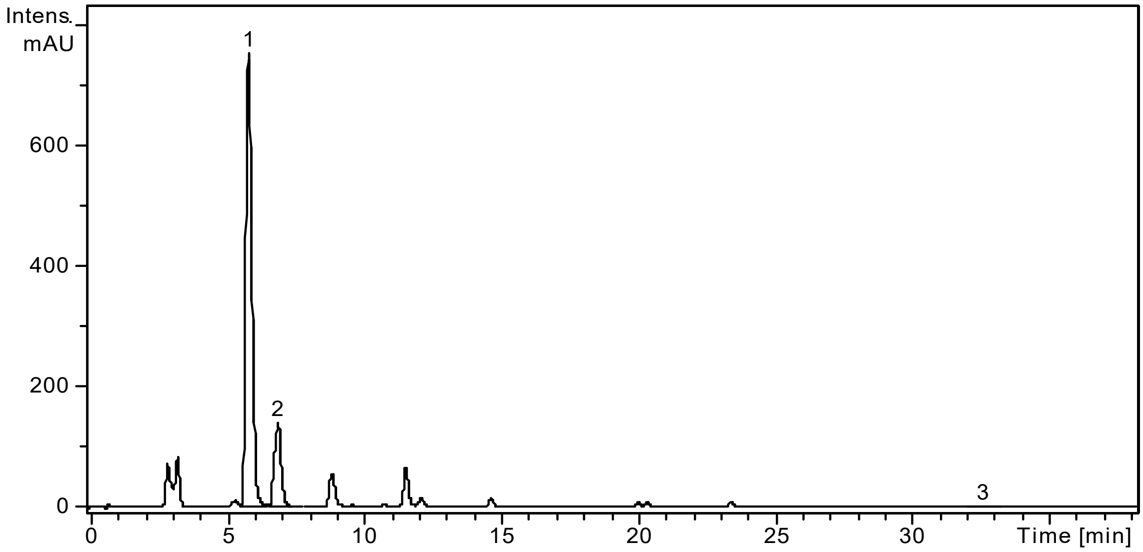

Figure S1. UV chromatogram of sample M: (1) chlorogenic acid, (2) kaempferol

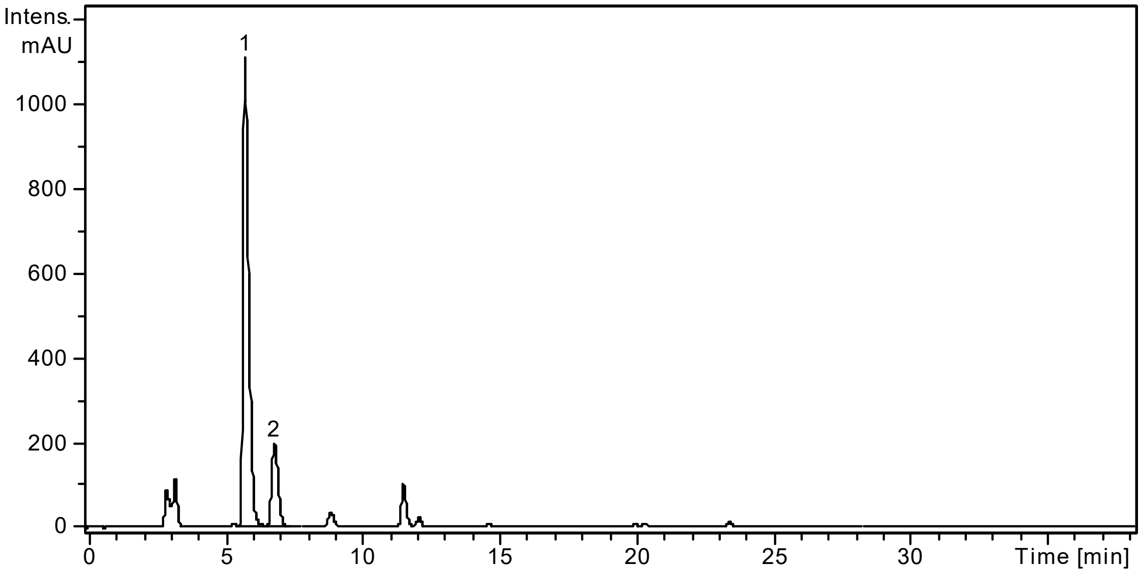

Figure S2. UV chromatogram of sample S20: (1) chlorogenic acid

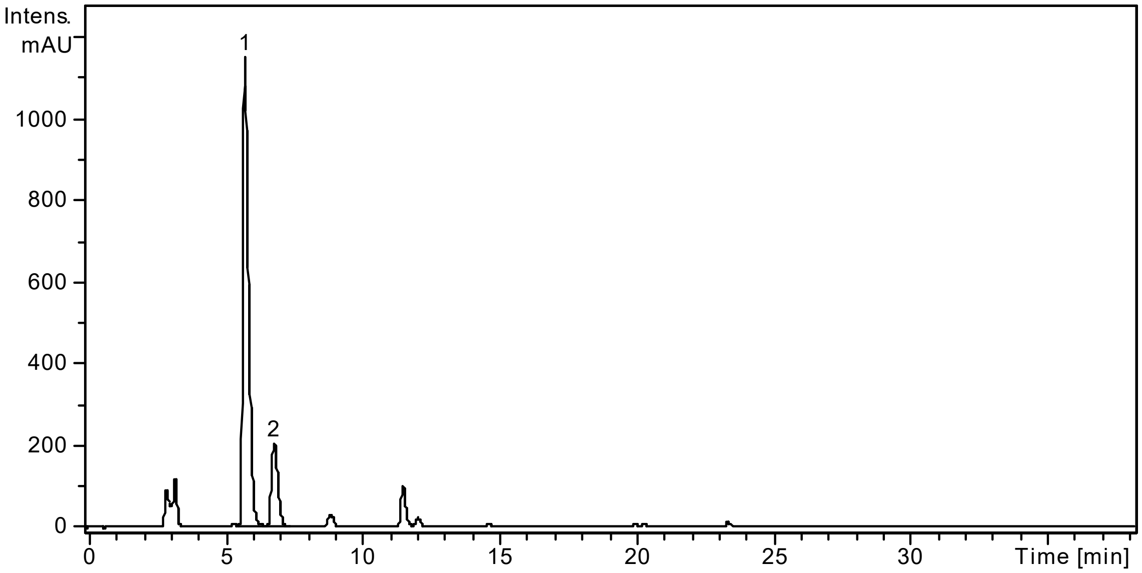

Figure S3. UV chromatogram of sample S40: (1) chlorogenic acid

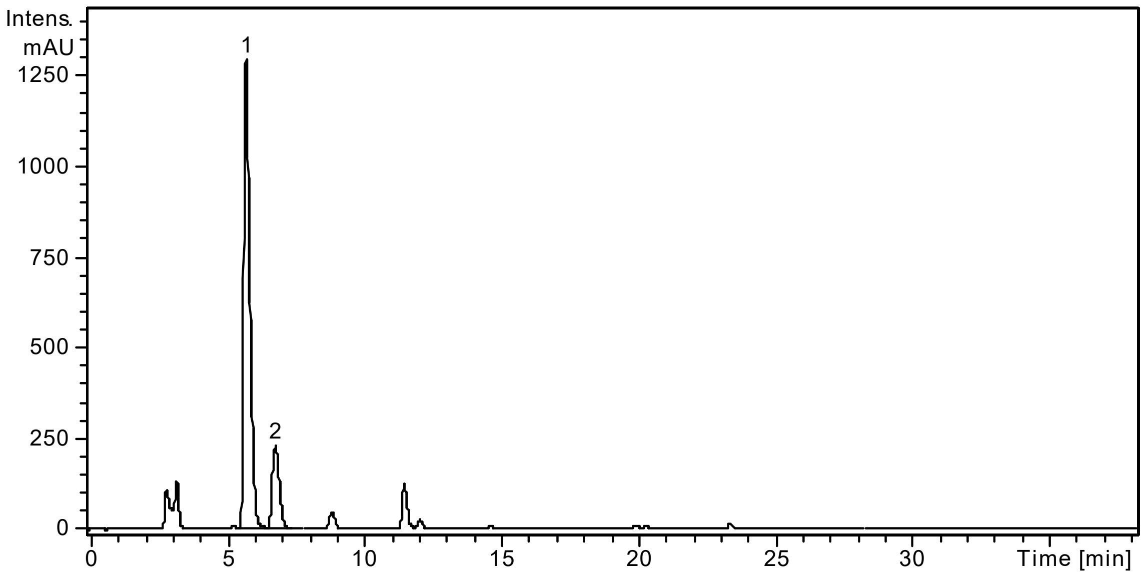

Figure S4. UV chromatogram of sample S60: (1) chlorogenic acid

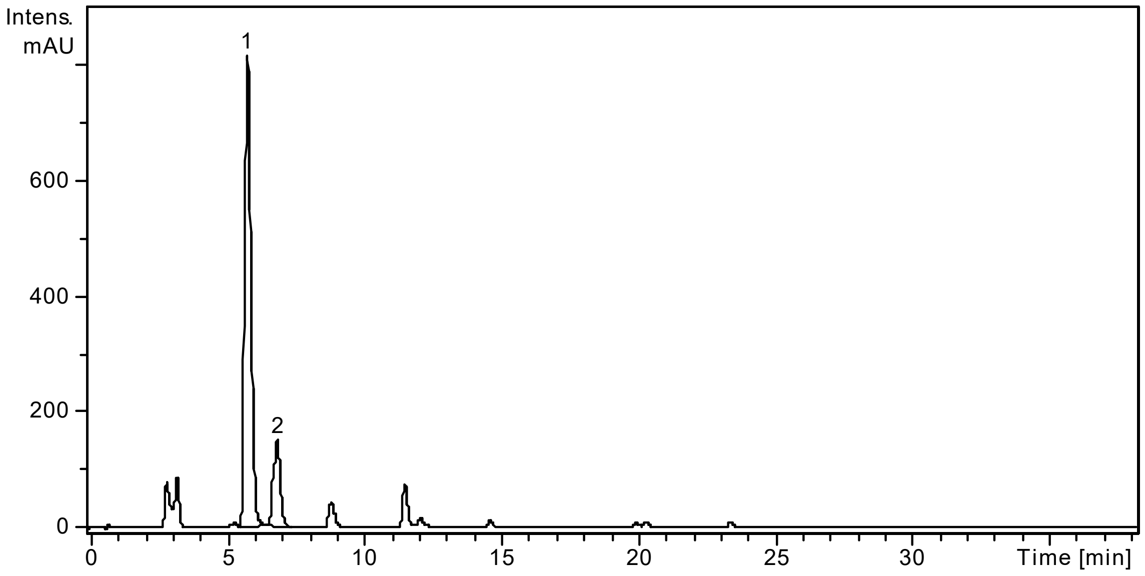

Figure S5. UV chromatogram of sample T24: (1) chlorogenic acid

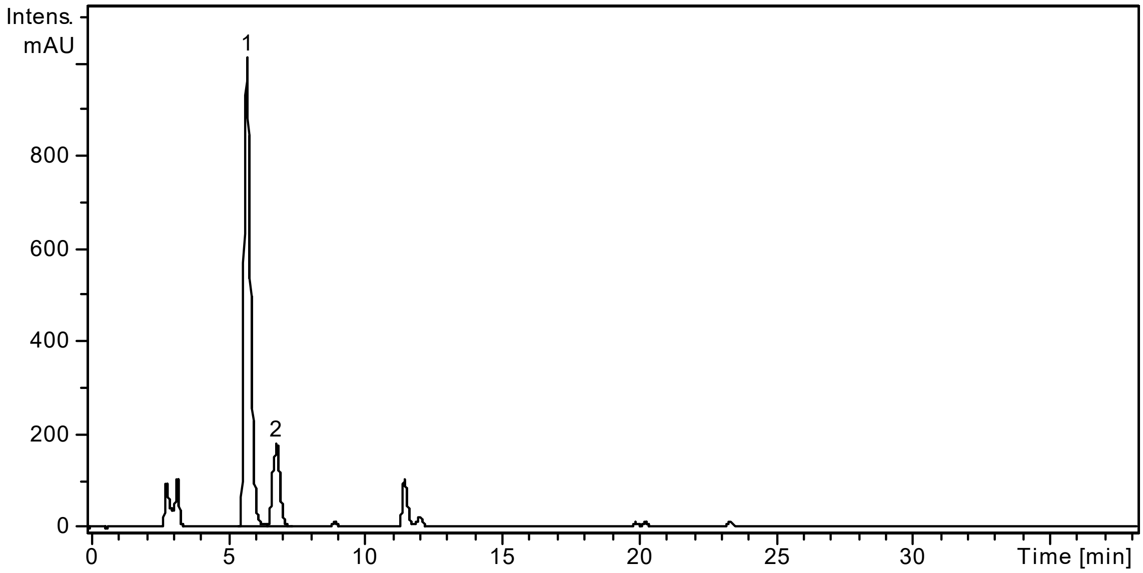

Figure S6. UV chromatogram of sample T44: (1) chlorogenic acid

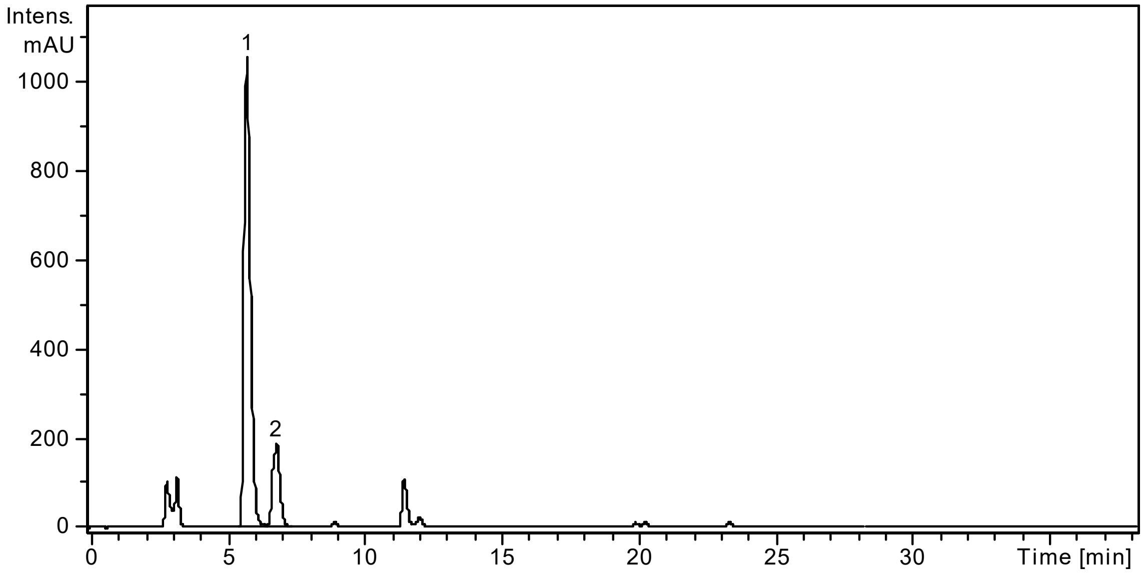

Figure S7. UV chromatogram of sample T46: (1) chlorogenic acid

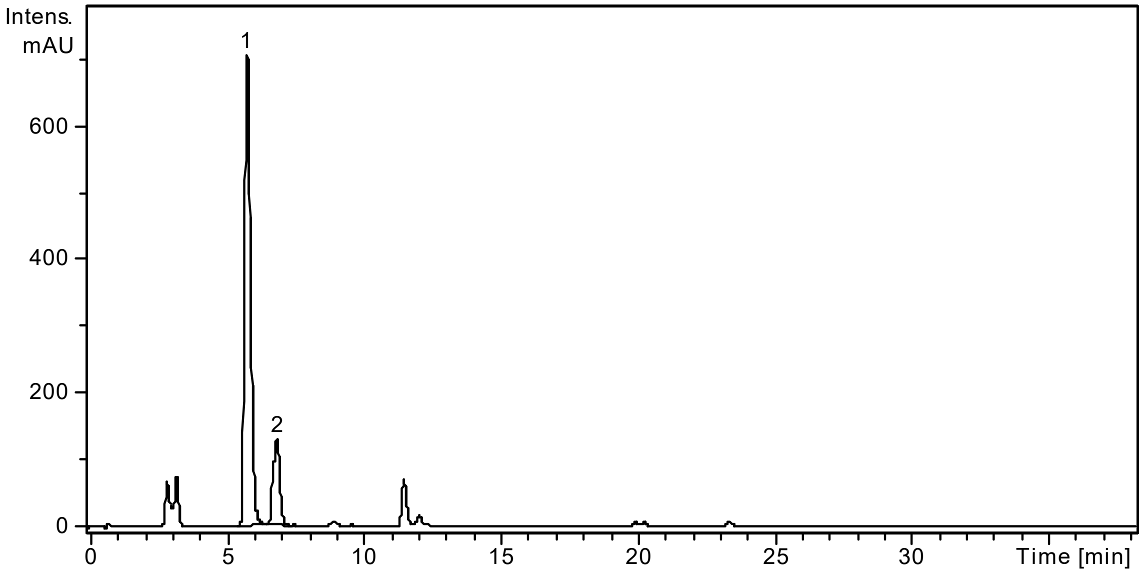

Figure S8. UV chromatogram of sample U23: (1) chlorogenic acid

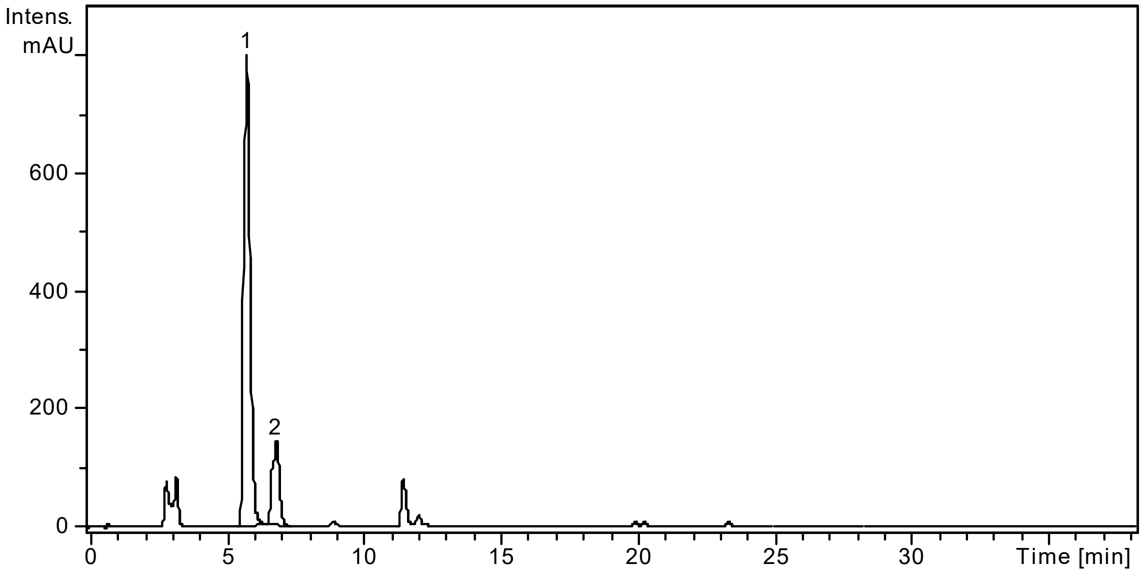

Figure S9. UV chromatogram of sample U34: (1) chlorogenic acid

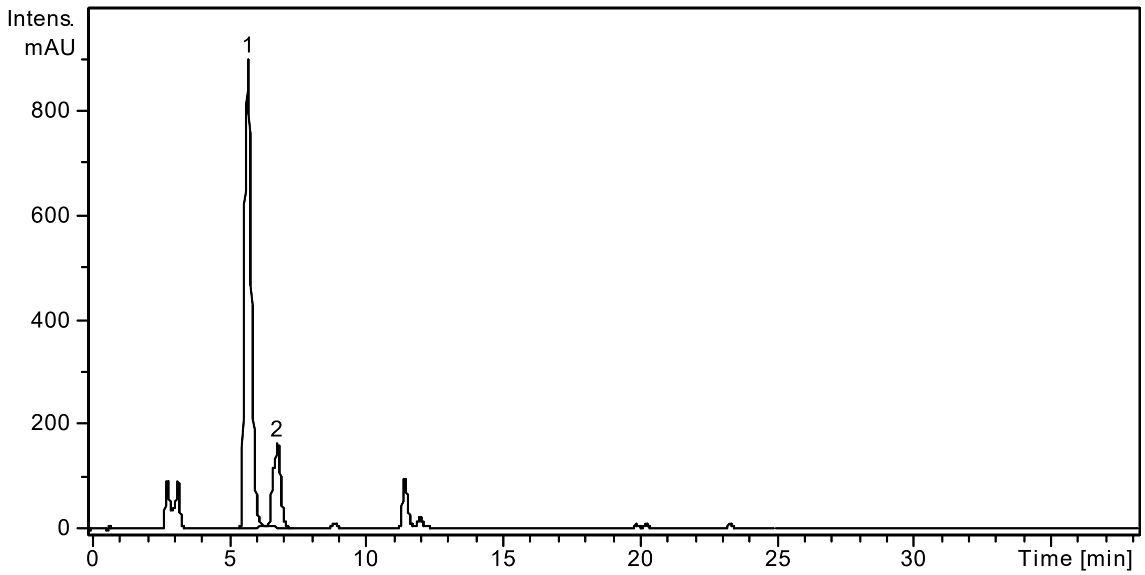

Figure S10. UV chromatogram of sample U35: (1) chlorogenic acid

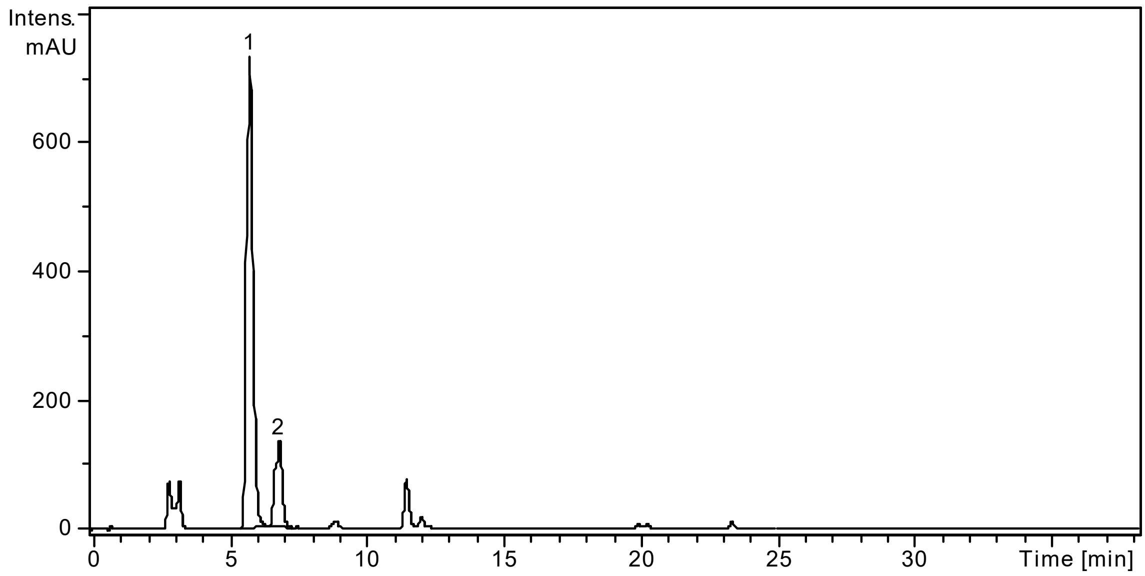

Figure S11. UV chromatogram of sample UT: (1) chlorogenic acid

Table S1 includes the analytical parameters of the database, such as retention time, precursor m/z, and specific ions used for the identification of the polyphenolic compounds, as well as the MS analysis type.

Table S1. MS analysis type and the characteristic ions from the mass spectrum of the analyzed polyphenolic compounds

No.	Bioactive compound name	MS analysis type	Retention time	Specific ions for identification<br>Ion [M-H]>Ions from spectra
1	Caftaric acid	MRM*	3.1	311>148.6, 178.6
2	Genistic acid	MRM	3.7	153>108.7
3	Caffeic acid	MRM	5.9	179.4>134.7
4	Chlorogenic acid	MRM	6.6	353.5>178.7, 190.7
5	4-O-caffeoylquinic acid	MRM	7.0	354.31>173, 179
6	p-coumaric acid	MRM	9.2	163> 118.7
7	Ferulic acid	MRM	12.4	193.2>133.7, 148.7, 177.6
8	Sinapic acid	MRM	14.7	223.4>148.6, 163.6, 178.7, 207.7
9	Vitexin	SIM**	18.0	431
10	Hyperoside	SIM	19.0	463
11	Vitexin 2-O-rhamnoside	SIM	19.4	577
12	Isoquercitrin	SIM	19.9	463
13	Rutin	SIM	20.4	609.1
14	Myricetin	SIM	21.1	317.1
15	Fisetin	SIM	22.8	285
16	Quercitrin	SIM	23.3	447.1
17	Kaemferitrin	SIM	25.6	577
18	Quercetol	SIM	26.8	301
19	Kaempferol 3-rhamnoside	SIM	27.4	431
20	Patuletin	SIM	28.7	331
21	Luteolin	SIM	29.2	285
22	Kaempferol	SIM	31.7	285
23	Apigenin	SIM	33.2	269.2

* MRM= multiple reaction monitoring

** SIM= single ion monitoring
